# Changes in corneal biomechanics in patients with glaucoma: a systematic review and meta-analysis

**DOI:** 10.1186/s12886-024-03443-4

**Published:** 2024-04-15

**Authors:** Xinru Li

**Affiliations:** Department of Ophthalmology, The First People’s Hospital of Yongkang Affiliated to Hangzhou Medical College, Yongkang, 321300 Zhejiang P. R. China

**Keywords:** Glaucoma, Corneal biomechanical properties, Meta-analysis

## Abstract

**Introduction:**

Corneal biomechanics has been implicated in a variety of ocular diseases. The purpose of this study was to evaluate the relationship between the glaucoma and corneal biomechanical properties, and exploring the value of corneal biomechanics in the diagnosis and follow-up of glaucoma diseases.

**Methods:**

We searched studies in PubMed, EMBASE, Web of Science and clinicaltrials.gov., as of October 8, 2022. Only English studies were included, without publication time limit. We also searched the reference lists of published reviews. This meta-analysis was conducted with random-effects models, we used mean difference(MD) to evaluate the outcome, and the heterogeneity was assessed with the I^2^ statistic. Subgroup analyses were performed under the appearance of high heterogeneity. We used 11 items to describe the characteristics of included studies, publication bias was performed through the Egger’s test. The quality assessment were evaluated by Newcastle–Ottawa Scale(NOS) items.

**Results:**

A total of 27 eligible studies were identified for data synthesis and assessment. The result of meta-analysis showed that in the comparison of included indicators, the corneal biomechanics values of glaucoma patients were statistically lower than those of normal subjects in a similar age range. The covered indicators included central corneal thickness(CCT) (MD = -8.34, 95% CI: [-11.74, -4.94]; *P* < 0.001), corneal hysteresis(CH)(MD = -1.54, 95% CI: [-1.88, -1.20]; *P* < 0.001), corneal resistance factor(CRF)( MD = -0.82, 95% CI: [-1.21, -0.44]; *P* < 0.001), and intraocular pressure(IOP)( corneal-compensated intraocular pressure (IOPcc): MD = 2.45, 95% CI: [1.51, 3.38]; *P* < 0.001); Goldmann-correlated intraocular pressure (IOPg): MD = 1.30, 95% CI: [0.41, 2.20]; *P* = 0.004), they all showed statistical difference. While the value of axial length(AL) did not show statistically different(MD = 0.13, 95% CI: [-0.24, 0.50]; *P* = 0.48).

**Conclusion:**

Corneal biomechanics are associated with glaucoma. The findings can be useful for the design of glaucoma screening, treatment and prognosis.

## Introduction

Glaucoma is gradually becoming the leading cause of irreversible blindness, and what’s more frightening is that it can be asymptomatic until it is severe. Previous surveys estimated that glaucoma might cause 12% of world blindness, more than 748 million dollars were annually spent on the glaucoma-related medical consultations, detection and surgeries [1, 2]. Glaucoma might be the second visual disorder after cataract [2]. Glaucoma is characterized by progressive degeneration of retinal ganglion cells, and the degeneration can change the appearance of optic disc and cause the loss of vision [3]. By the progression of disease, some different signs and symptoms gradually appeared, including raised IOP, haloes around lights, cloudy cornea, pain( not the typical characteristic in primary open-angle glaucoma(POAG)), visual field loss and optic disc changes [4]. Unfortunately, glaucoma as a multifactorial disease, no effective treatment has been found to reverse the visual damage caused by glaucoma. Fortunately, we can control the progression of glaucoma through early detection, early diagnosis and early intervention treatment. Therefore, the timing of diagnosis and surgical intervention is important to the visual prognosis.

The accurate diagnosis of glaucoma requires a number of subjective and objective ophthalmic examinations [5]. Conventional evaluation of glaucoma disease progression is mainly based on the examination of visual field, optic disc and blood vessel changes of the fundus. Ocular hypertension is a common symptom in glaucomatous patients. In other words, IOP measurement is important in the diagnosis of glaucoma. IOP as an important diagnostic basis of glaucoma, its determination depends highly upon the corneal biomechanical characteristics. And the IOP is affected by corneal biomechanical factors, such as CCT, elasticity, hydration, hysteresis and rigidity [6]. The corneal biomechanical properties are important to the management of some ocular diseases, and they also can predict or assess the surgical responses [7]. New techniques and devices allow experts to do an accurate diagnosis and evaluation whenever before or after therapy.

The methods of biomechanical assessment are various, the machines include Ocular Response Analyzer (ORA), Corneal Visualization Scheimpflug Technology instrument (Corvis ST), Air-puff OCT, and Optical Coherence Elastography (OCE). These techniques monitor the bidirectional deformation of the cornea through two applanation points. Despite having the common mechanism, these techniques are different in their applied forces and analytical methods [7]. The machines used in our research are mainly ORA and Corvis ST.

Corneal structure and mechanical behavior, are they associated with glaucoma? Or are they affected during the progression of glaucoma? If the answer is definite, can we diagnose glaucoma or evaluate glaucoma status through the changes in corneal biomechanics? To sum up, we try to analyze the relationship between glaucoma and corneal biomechanics through this meta-analysis. And if the result is meaningful, it can provide greater clinical methods through biomechanical analysis and support more personalized medical decision-making.

## Methods

### Data source and search strategy

We searched relevant content in the databases of PubMed, EMBASE, Web of Science and clinicaltrials.gov. No date is limited, and the publication is limited to English language. We performed this search on October 8, 2022. Search syntax was (corneal biomechanics OR corneal biomechanical ocular response analyzer OR corneal hysteresis OR corneal resistance factor) AND (glaucoma). Search fields were [Title/Abstract] in PubMed and EMBASE, [Topic] in Web of Science. To prevent omissions, we also searched in the reference lists of included studies and related reviews. Two reviewers screened the articles independently, the third reviewer eliminated duplicate articles. And the disputes were resolved by the supervisor.

### Study selection: inclusion and exclusion criteria

Inclusion criteria:

1. participants fulfilled the diagnostic criteria for glaucoma, no matter the types of glaucoma( optic nerve morphology characteristic of glaucomatous optic neuropathy, glaucomatous appearance of the optic disc with corresponding visual field changes);
2. without age limitation, glaucoma and normal subjects were age-matched;
3. without ethnicity or country limitation;
4. without gender limitation;
5. having sufficient visual acuity for fixation;
6. concurrent or prior use of topical medication was not excluded;
7. normal groups required no signs and evidence of glaucoma, non-glaucomatous optic nerve pathology or retinal pathology.

Exclusion criteria:

1. corneal pathologic conditions which might affect the measurement results;
2. prior refractive or corneal surgery;
3. amblyopia, strabismus, other systemic or ocular disorders, including intraocular surgeries or refractive surgeries;
4. children subjects have a family history of glaucoma.

### Data collection and quality assessment

Two reviewers independently screened titles and abstracts to obtain eligible articles. When more than one report used the same data and measured items, only the latest report was included to avoid duplicate counting. If it was the same set of data, but it was different types of subjects or indicators, we still included to research. We extracted relevant data regarding study characteristics( author, study design, country, the machine type of measurement), patient characteristics(ethnicity, gender, age, the number of included eyes, the type of glaucoma, treatment history, IOP_GAT_), and corresponding outcomes. Disagreements between individual judgments were resolved through a discussion with the supervisor. The quality of included studies was assessed by NOS, the more stars indicated higher scores and represented studies of higher quality.

### Statistical analysis

We performed comparisons using Review Manager(version 5.4; Cochrane Collaboration) and Stata Software(version 16.0; Stata Corp LP, College Station, TX). We conducted analyses for the correlation between glaucoma and corneal biomechanics, calculated the MD and 95% confidence intervals(CIs) for various groups in AL, CCT, CH, CRF, IOPcc and IOPg. The measurement data was presented as the MD and standard deviation(SD), each effect size was expressed as CI. All the values were analyzed statistically using the random-effects model. The heterogeneity was statistically assessed by I^2^ statistics in studies. The I^2^ statistic of > 50% indicated high heterogeneity, 25%-50% indicated moderate heterogeneity, and < 25% indicated low heterogeneity. Subgroup analyses were performed if high heterogeneity was observed. Sensitivity analyses were performed to evaluate whether the results were affected by the single study. Publication bias was performed through Egger’s test. The study characteristics were assessed by 11 items. If *P* value is less than 0.05, the difference between groups is statistically significant.

## Results

### Literature search

**Fig. 1:**
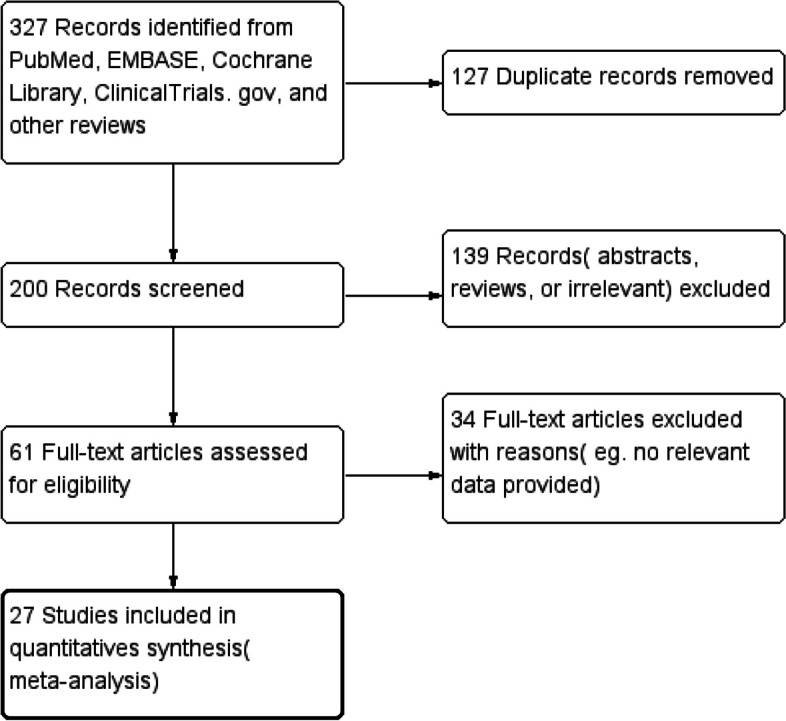
PRISM flow diagram of the literature search process

We identified 327 studies through literature searches, through an initial screening of duplicate studies, 200 relevant studies were included. Sixty-one studies were included after removing the studies which could not fulfill the inclusion criteria. After excluding the papers which could not provide the relevant or valid data, excluding the studies that we were unable to get original data, 27 observational studies constituted the data for analyzing [8–34] (Fig. 1). A total of 5935 eyes were included. The characteristics of the included studies were presented in Table 1. There were 8 kinds of glaucoma in the included studies. Different machines were used to measure the related indicators, ORA and Corvis ST were the main measurement machines in the included studies.

**Table 1:** Characteristics of Included Studies in the meta-analysis

Author, Year	Study	Country	Ethnicity	Gender	Age(controls/patients)	Normal sample size(eye)	Experimental sample size(eye)	The type of glaucoma	Whether patients were treated for glaucoma	Machine	IOP_GAT_(mmHg)
Morales, 2021 [8]	Observational, cross-sectional, study	Spain	-	M/F	(21.45 ± 9.94)/(18.60 ± 11.65)	40	50	PCG	-	ORA	18.18 ± 3.94
Sullivan, 2008 [9]	Observational, cross-sectional study	The United States	Whites, Hispanics, blacks, native Americans	-	(64.5 ± 12.9)/(71.9 ± 10.0)	71	99	GLC	-	ORA	-
Hocaoglu, 2020 [10]	Observational, cross-sectional study	Turkey	-	M/F	(55.43 ± 8.65)/(62.96 ± 8.15)	133	68	POAG	Yes	ORA	16.65 ± 5.42
Fujishiro, 2020 [11]	Observational, cross-sectional study	Japan	-	M/F	(31.5 ± 5.1)/(62.9 ± 10.3)	35	104	POAG	-	Corvis ST, ORA	-
Aoki, 2021 [12]	Retrospective, cross-sectional study	Japan	-	-	(69.4 ± 13.9)/(69.1 ± 13.4)	68	68	POAG	Yes	Corvis ST, ORA	12.9 ± 3.2
Park, 2018 [13]	Retrospective, cross-sectional study	Korea	-	M/F	(56.35 ± 10.46)/NTG early: (53.92 ± 12.03)/advanced: (62.38 ± 11.96)	93	95	NTG	No	ORA	NTG early: 15.10 ± 3.18/ advanced: 15.17 ± 2.99
Cankaya, 2012 [14]	Observational, cross-sectional study	Turkey	-	M/F	(68.4 ± 5.7)/(70.2 ± 7.3)	102	78	EXG	-	ORA	16.3 ± 4.1
Yazgan, 2015 [15]	Observational, cross-sectional study	Turkey	-	M/F	(67.83 ± 6.75)/(73.50 ± 5.36)	45	30	PEXG	-	ORA	15.7 ± 4.02
Detry, 2011 [16]	Observational, cross-sectional study	Belgium	-	M/F	(58.0 ± 14.0)/(70.0 ± 11.0)	24	108	POAG	-	ORA	17.0 ± 4.3
Perucho, 2016 [17]	Observational, cross-sectional study	Spain	Caucasian	-	(18.07 ± 11.34)/(19.18 ± 11.45)	103	118	PCG	-	ORA	18.32 ± 5.13
Perucho, 2017 [18]	Observational, cross-sectional study	Spain	-	M/F	(5.20 ± 3.25)/(5.64 ± 2.85)	66	94	PCG	-	ORA	-
Gatzioufas, 2013 [19]	Prospective, observational study	German	-	M/F	(14.2 ± 3.6)/(13.6 ± 4.8)	40	40	PCG	-	ORA	
Morita, 2012 [20]	Observational, cross-sectional study	Japan	-	M/F	(57.7 ± 12.1)/(59.1 ± 12.3)	83	83	NTG	-	ORA	14.0 ± 2.2
Costin, 2014 [21]	Prospective, observational study	Finland	-	-	(56.5 ± 5.7)/(63.6 ± 12.1)	15	13	POAG	-	ORA	14.5 ± 3.6
Beyazyıldız, 2014 [22]	Observational cross-sectional study	Turkey	-	M/F	Control: (51.2 ± 11.6)/EXG: (68.6 ± 8.5)/POAG: (58.9 ± 10.7)	50	46/66	EXG/POAG	-	ORA	EXG: (16.5 ± 4.1)/POAG: (16.4 ± 4.2)
Mangouritsas, 2009 [23]	Prospective, observational study	Greece	-	M/F	(59.2 ± 14.2)/(62.4 ± 9.8)	74	108	POAG	-	ORA	16.38 ± 2.73
Narayanaswamy, 2011 [24]	Prospective observational study	Singapore	-	M/F	Control: (54.7 ± 8.5)/PACG: (67.1 ± 9.8)/POAG: (64.6 ± 10.5)	150	131/162	PACG/POAG	-	ORA	(16.4 ± 0.8)/(14.4 ± 0.5)
Kaushik, 2012 [25]	Prospective observational study	India	-	M/F	Unclear(> 18 years of age)	71	59/36	PACG/POAG	-	ORA	PACG(16.2 ± 3.9)/POAG: (23.6 ± 12.4)
Shin, 2015 [26]	Prospective, cross-sectional study	Korea	-	M/F	(49.0 ± 16.07)/(52.24 ± 14.48)	89	97	NTG	-	ORA	GAT: (14.94 ± 3.27)/ICare: (14.71 ± 3.19)
Ayala, 2011 [27]	Retrospective, cross-sectional study	Sweden	-	M/F	Control: (67 ± 9)/POAG: (62 ± 13)/PXSG: (71 ± 9)	30	30/30	POAG/PXSG	-	ORA	POAG: (16.4 ± 4.6)/PXSG: (17.5 ± 5.6)
Detry, 2012 [28]	Observational cross-sectional study	Belgium	African, Caucasian	M/F	African: (43.9 ± 11.4)/(53.8 ± 12.7) Caucasian: (58.4 ± 14.7)/(70.6 ± 9.2)	55	59	POAG	-	ORA	African: (18.0 ± 5.0) Caucasian: (16.4 ± 3.7)
Grise, 2012 [29]	Retrospective, cross-sectional study	France	-	-	Control: (57.5 ± 5.9)/NTG: (56.1 ± 5.1)/POAG: (59.9 ± 4.9)	44	28/75	NTG/POAG	-	ORA	NTG: (13.0 ± 2.63)/POAG: (18.0 ± 4.42)
Morales, 2022 [33]	Observational cross-sectional study	Spain	-	M/F	Unclear(> 18 years of age)	40	40	PCG	-	ORA	-
Jung, 2020 [31]	Retrospective cross-sectional study	Korea	-	M/F	Control: (56.19 ± 12.45)/POAG: (55.13 ± 15.65)/EXG: (57.77 ± 13.00)	61	46/54	POAG/NTG	32/38^a^	Corvis ST	-
Miki, 2020 [30]	Retrospective cross-sectional study	Japan	-	-	(56.4 ± 13.2)/(52.7 ± 14.6)	35	35	NTG	-	Corvis ST	15.6 ± 2.8
Hussnain, 2015 [32]	Retrospective, cross-sectional study	The United States	-	-	(61.59 ± 16.56)/(70.73 ± 11.33)	1418	322	POAG	-	ORA	-
Reznicek, 2013 [34]	Prospective observational study	German	-	-	Control: (55.4 ± 15.5)/ OAG: (63.1 ± 13.9)	36	142/106/14/22	OAG/POAG/NTG/PEXG	-	Corvis ST	OAG: (15.4 ± 6.1)/POAG: (16.5 ± 7.2)/NTG: (11.1 ± 1.5)/PEXG: (13.9 ± 3.5)

-: not mentioned

*ORA* Ocular Response Analyzer, *Corvis ST* Corneal Visualization Scheimpflug Technology instrument, *IOP*_*GAT*_ intraocular pressure of Goldmann applanation tonometry, *M/F* including male and female, *PCG* primary congenital glaucoma, *GLC* glaucoma, *OAG* open-angle glaucoma, *POAG* primary open-angle glaucoma, *EXG* exfoliative glaucoma, *PEXG* pseudoexfoliative glaucoma, *NTG* normal-tension glaucoma, *PACG* primary angle-closure glaucoma, *PXSG* pseudoex-foliative glaucoma

^a^Jung et al. showed that 32 and 38 patients respectively used the prostaglandin analogues in the POAG and EXG group in their study

### Study characteristics

The study characteristics were shown in Table 1. There were 27 included studies involving different ages, > 18 years of age(*n* = 24), < 18 years of age(*n* = 2), and one study that we did not get the clear age boundary information(*n* = 1). Most studies did not mention the ethnicity of included groups(*n* = 24), only four publications labeled the ethnicity. Eighteen publications had both male and female volunteers and patient subjects, and 9 publications did not mention the sex of the included subjects. Only three studies clearly stated that the included glaucoma patients were all used the topical medications, one study stated the specific number of people who had concurrent or prior use in the glaucoma groups, and none of the remaining groups explicitly described the use of medication. The type of glaucoma was various in different studies, including open-angle glaucoma(OAG, *n* = 1), POAG(*n* = 15), primary congenital glaucoma(PCG, *n* = 5), normal-tension glaucoma(NTG, *n* = 7), exfoliative glaucoma(EXG, *n* = 4), primary angle-closure glaucoma(PACG, *n* = 1), pseudoex-foliative glaucoma(PXSG, *n* = 1), glaucoma(GLC, not distinguished the glaucoma types, *n* = 1), one study was never stated the type of glaucoma and some studies were included two kinds of glaucomatous types. The 27 studies were chosen from 21 laboratories in thirteen countries. There were 22 publications used ORA machines to measure the related corneal index, and 3 publications used Corvis ST machines.

### Comparison analysis

#### Comparison of AL values between glaucoma patients and normal subjects

**Fig. 2:**
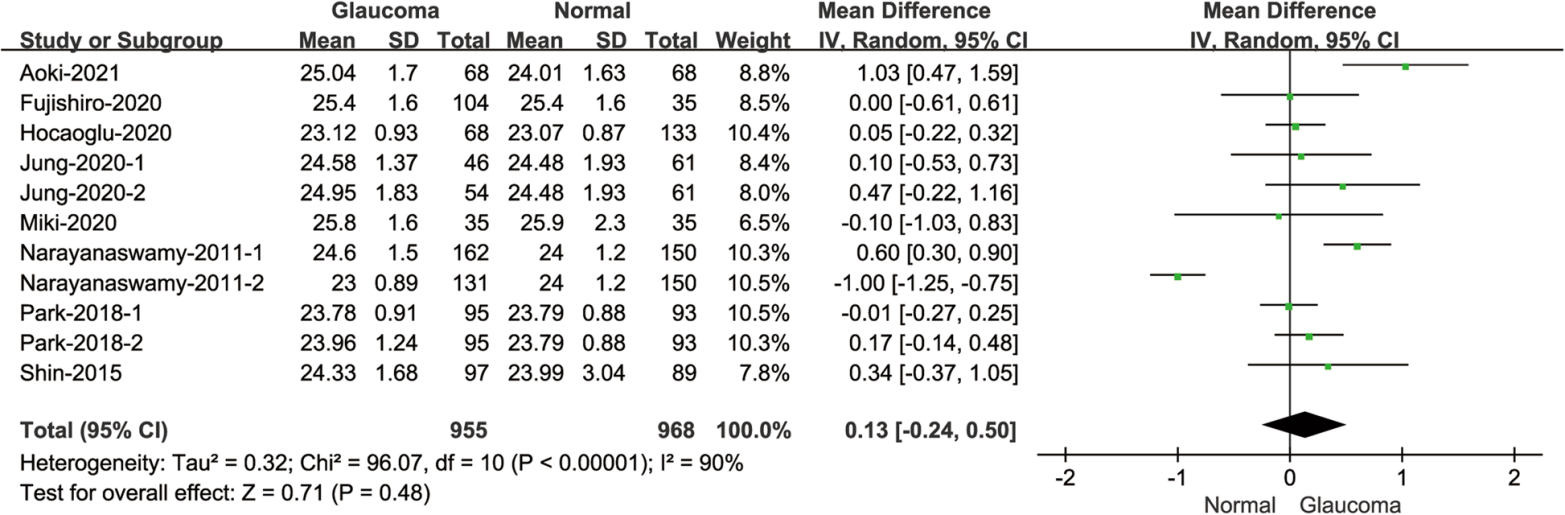
Forest plot of comparison in AL

Eight articles were included to compare the AL of glaucoma patients (Fig. 2), with different types of glaucoma groups in some studies, there were 11 groups included to be statistically analyzed. The AL values of glaucoma patients did not show statistical difference(MD = 0.13, 95% CI: [-0.24, 0.50]; *P* = 0.48). And the data of AL values comparisons was highly heterogeneous(I^2^ = 90%; *P* < 0.001).

#### Comparison of CCT between glaucoma patients and normal subjects

**Fig. 3:**
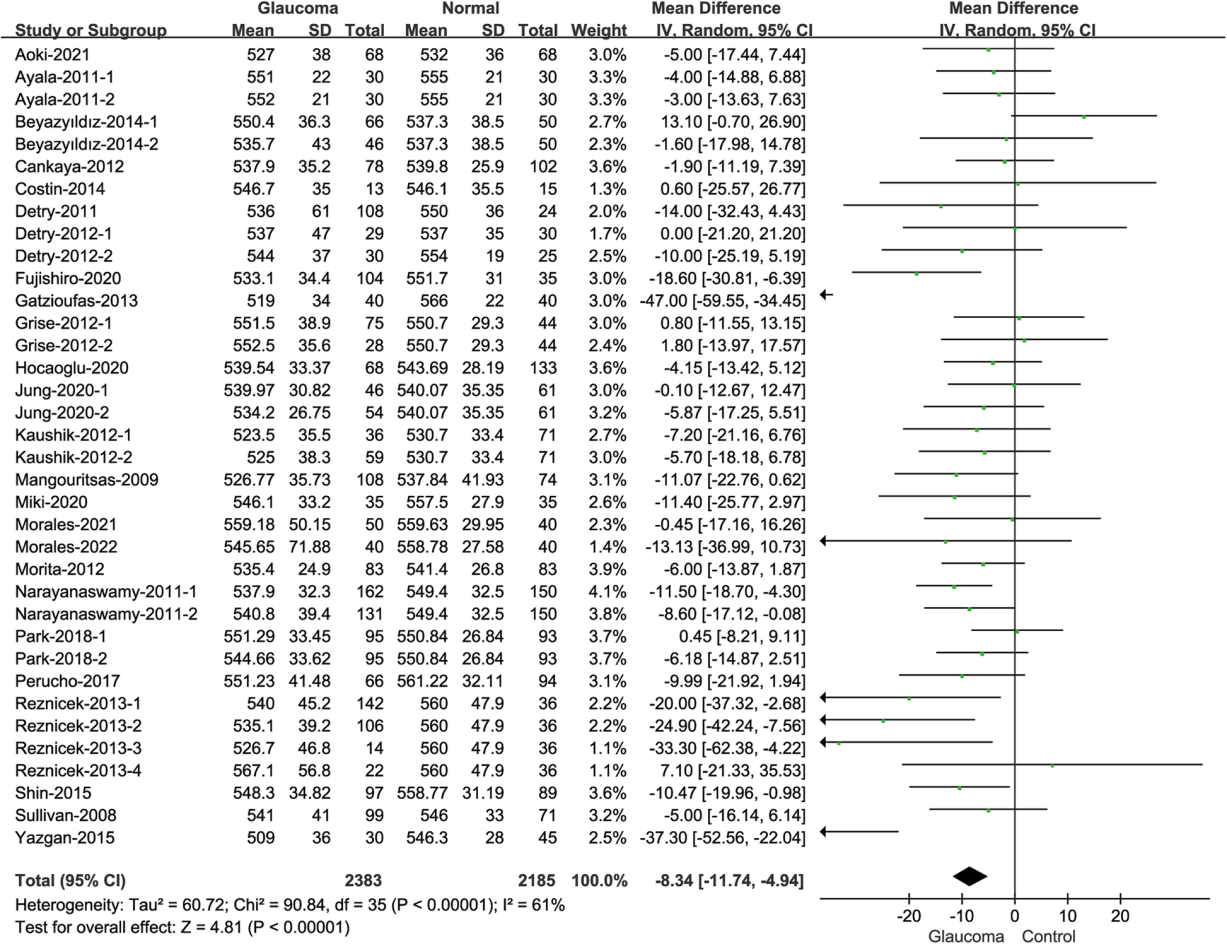
Forest plot of comparison in CCT

A comparison was conducted on the CCT values of glaucoma patients and normal subjects, as by twenty-five of the 27 studies (Fig. 3). The heterogeneity was statistically high(I^2^ = 61%; *P* < 0.001). The results showed that the CCT values of glaucoma patients were statistically higher than the normal subjects(MD = -8.34, 95% CI: [-11.74, -4.94]; *P* < 0.001).

#### Comparison of CH between glaucoma patients and normal subjects

**Fig. 4:**
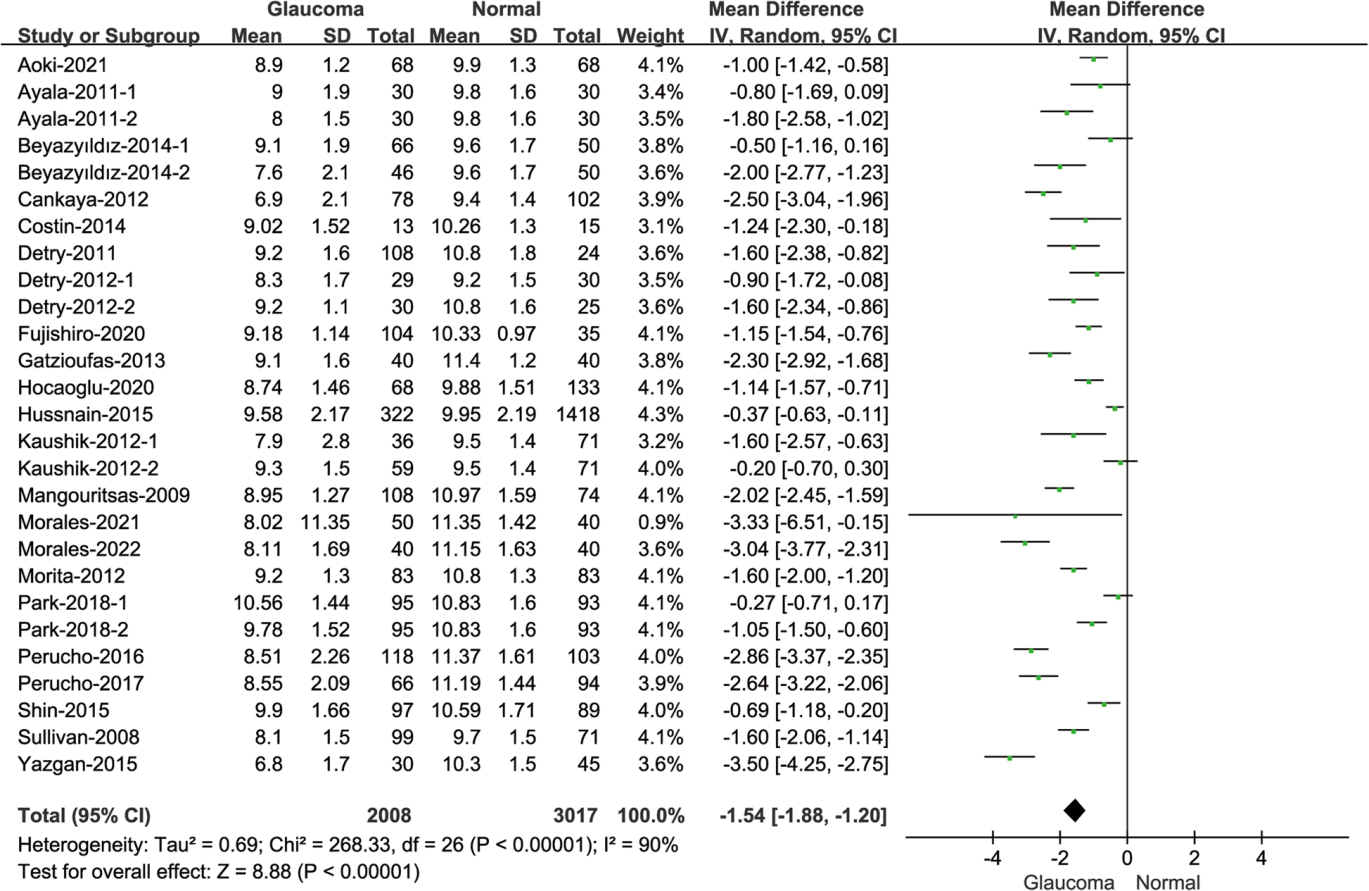
Forest plot of comparison in CH

Twenty-two of included studies reported the changes of CH in different subjects (Fig. 4). The comparison between glaucoma patients and normal subjects showed that the CH values of glaucoma patients were statistically lower than normal subjects(MD = -1.54, 95% CI: [-1.88, -1.20]; *P* < 0.001). And the heterogeneity was considerable((I^2^ = 90%; *P* < 0.001).

#### Comparison of CRF between glaucoma patients and normal subjects

**Fig. 5:**
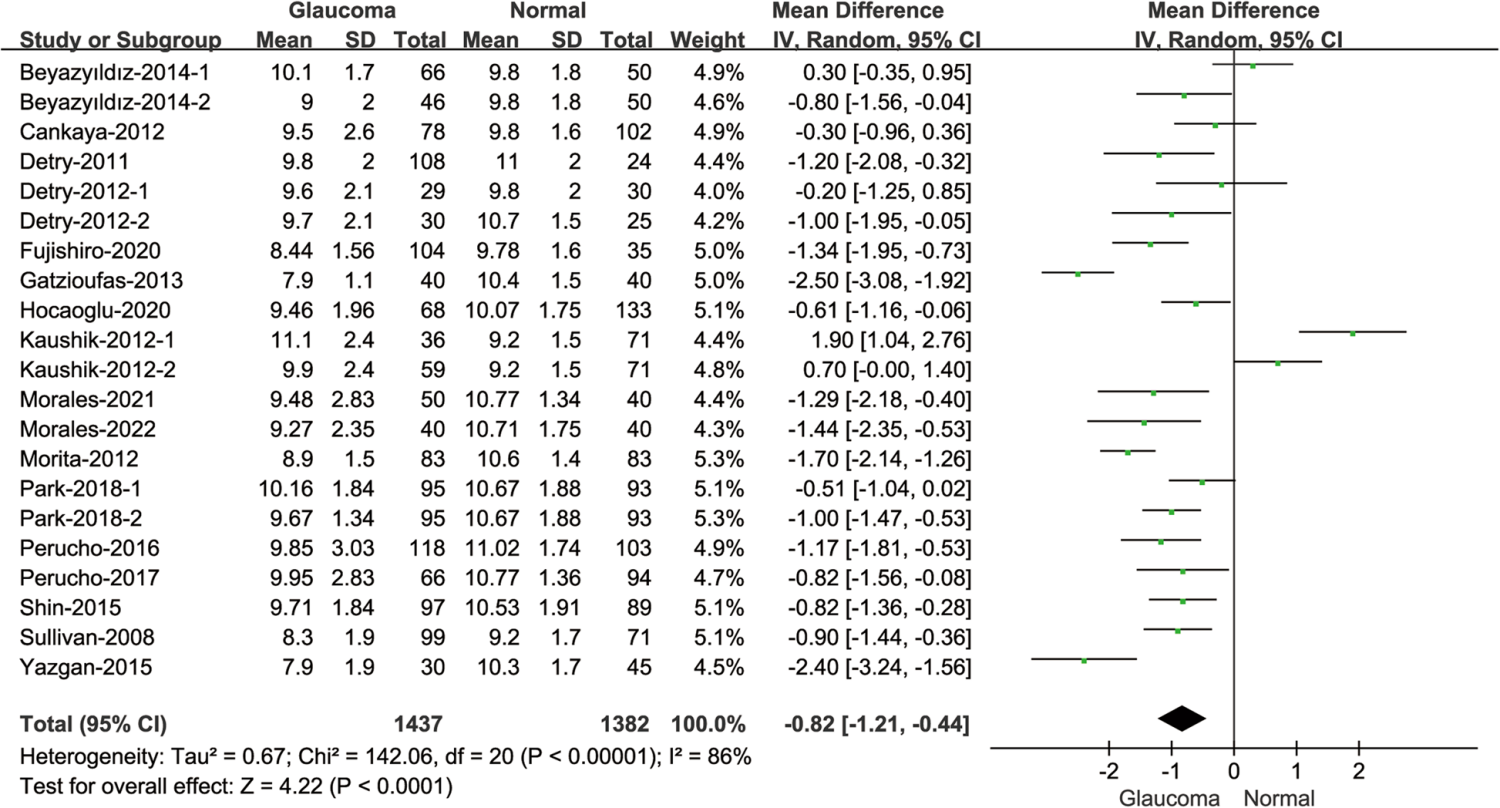
Forest plot of comparison in CRF

There were 17 studies participated in the comparison of CRF values (Fig. 5). The heterogeneity was high(I^2^ = 86%; *P* < 0.001). Comparing the CRF values between glaucoma patients and normal subjects, the result showed that the CRF of glaucoma patients was statistically lower than normal subjects(MD = -0.82, 95% CI: [-1.21, -0.44]; *P* < 0.001).

#### Comparison of IOPcc and IOPg between glaucoma patients and normal subjects

**Fig. 6:**
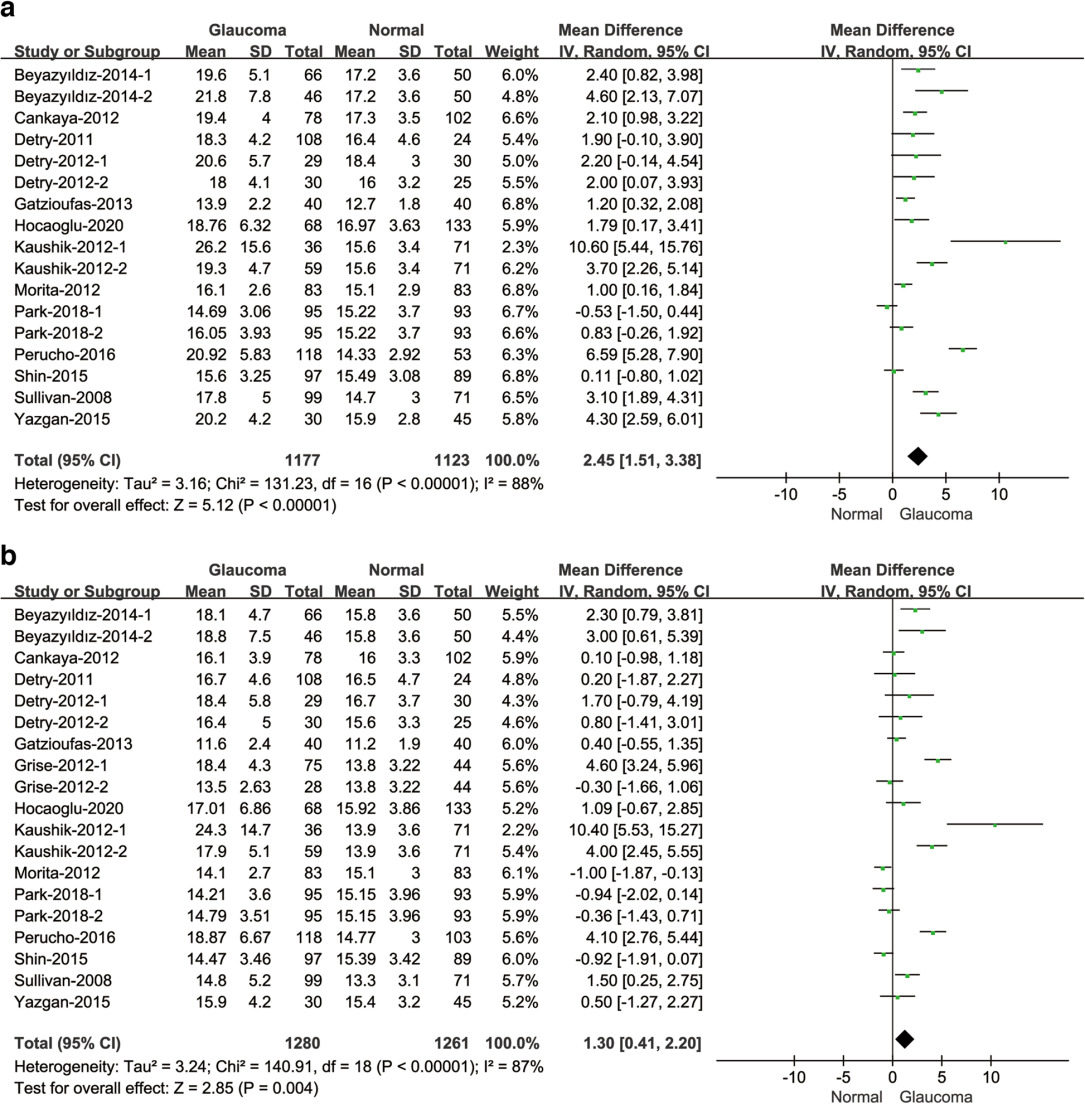
Forest plot of comparison in IOPcc (**a**) and forest plot of comparison in IOPg (**b**)

There were 13 studies reported the comparison of IOPcc values and 14 studies reported the comparison of IOPg values in different subjects (Fig. 6). Comparing the IOPcc values, the result demonstrated that there was a high heterogeneity(I^2^ = 88%; *P* < 0.001), and the IOPcc of glaucoma patients was higher than normal subjects(MD = 2.45, 95% CI: [1.51, 3.38]; *P* < 0.001). The heterogeneity of IOPg values was high as well(I^2^ = 87%; *P* < 0.001), the IOPg values of normal subjects were lower than glaucoma patients(MD = 1.30, 95% CI: [0.41, 2.20]; *P* = 0.004).

### Subgroup analysis

We performed subgroup analyses of the possible factors that might cause the high heterogeneity (Table 2). We conducted subgroup analyses from four aspects, including age, treatment history, the type of glaucoma and machine. For the CCT values, the subgroup analysis of age delivered that ‘ ≥ 18 years old’ could slightly reduce the heterogeneity(I^2^ = 37%), while the group of ‘ < 18 years old’ did not decrease the heterogeneity. In terms of treatment history, both ‘Used medicine’ and ‘Not used medicine’ groups showed low heterogeneity(I^2^ = 0%; I^2^ = 11%), however the ‘Not mentioned’ group which not stated the history of treatments still showed high heterogeneity(I^2^ = 65%). For the type of glaucoma group, only the ‘PACG’ group showed low heterogeneity(I^2^ = 0%). For the type of machine group, the heterogeneity of ‘Corvis ST’ group was moderate(I^2^ = 45%).

**Table 2:** Subgroup analyses of CCT, CH and CRF

						Heterogeneity			
		No	MD(95%CI)		Q	I^2^	P_Q_	χ^**2**^	P
a. Subgroup analysis of CCT									
Age	≥ 18 years old < 18 years old	4328 240	-6.89(-9.61, -4.17) -28.44(-64.71, 7.83)		52.20 17.55	37% 94%	0.02 0.000	4.964 1.54	0.000 0.12
Whether patients were treated for glaucoma	Used medicine Not used medicine Not mentioned	559 376 3633	-3.94(-9.51, 1.64) -2.86(-9.35, 3.64) -9.60(-13.71, -5.49)		0.50 1.12 83.43	0% 11% 65%	0.92 0.29 0.000	1.38 0.86 4.58	0.17 0.39 0.000
	OAG	178	-20.00(-37.32, -2.68)		-	-	-	2.26	0.02
	POAG NTG	1895 1035	-6.59(-10.86,-2.32) -5.97(-10.00, -1.94)		22.56 7.85	38% 11%	0.07 0.35	3.03 2.90	0.002 0.004
The type of glaucoma	PCG GLC	410 170	-18.11(-40.65, 4.43) -5.00(-16.14, 6.14)		25.99 -	88% -	0.000 -	1.57 0.88	0.12 0.38
	EXG PACG PXSG	409 411 60	-9.48 (-28.43,9.47) -7.68(-14.72, -0.64) -3.00(-13.63, 7.63)		17.46 0.14 -	83% 0% -	0.000 0.71 -	0.98 2.14 0.55	0.33 0.03 0.58
The type of machine	ORA Corvis ST ORA and Corvis ST	3573 720 275	-7.48(-11.42,-3.55) -11.34(-19.87, -2.80) -11.85(-25.18, 1.47)		76.00 10.90 2.34	66% 45% 57%	0.000 0.09 0.13	3.73 2.60 1.74	0.000 0.009 0.08
b. Subgroup analysis of CH									
Age	≥ 18 years old < 18 years old Not clearly defined	4564 240 221	-1.39(-1.72, -1.06) -2.48(-2.90, -2.06) -2.86(-3.37, -2.35)	196.47 0.61 -		88% 0% -	0.000 0.43 -	8.30 11.46 10.93	0.000 0.000 0.000
Whether patients were treated for glaucoma	Used medicine Not used medicine Not mentioned	337 376 4312	-1.07(-1.37, -0.77) -0.66(-1.42, 0.11) -1.68(-2.07, -1.28)	0.21 6.01 238.11		0% 83% 91%	0.65 0.01 0.000	6.95 1.69 8.27	0.000 0.09 0.000
	POAG NTG	2955 728	-1.14(-1.49,-0.79) -0.91(-1.49, -0.33)	53.11 21.04		79% 86%	0.000 0.000	6.40 3.06	0.000 0.002
The type of glaucoma	PCG GLC	631 170	-2.71(-3.01, -2.41) -1.60(-2.06, -1.14)	3.00 -		0% -	0.56 -	17.88 6.86	0.000 0.000
	EXG PACG PXSG	351 130 60	-2.66 (-3.45, -1.88) -0.20(-0.70, 0.30) -1.80(-2.58, -1.02)	7.98 - -		75% - -	0.02 - -	6.64 0.78 4.50	0.000 0.44 0.000
The type of machine	ORA Corvis ST ORA and Corvis ST	4750 - 275	-1.59(-1.96,-1.21) - -1.08(-1.37, -0.80)	265.29 - 0.26		91% - 0%	0.000 - 0.61	8.24 - 7.42	0.000 - 0.000
c. Subgroup analysis of CRF									
Age	≥ 18 years old < 18 years old Not clearly defined	2358 240 221	-0.71(-1.10, -0.31) -1.68(-3.32, -0.03) -1.17(-1.81, -0.53)	109.33 12.40 -		84% 92% -	0.000 0.000 -	3.49 2.00 3.57	0.000 0.05 0.000
Whether patients were treated for glaucoma	Used medicine Not used medicine Not mentioned	201 376 2242	-0.61(-1.16, -0.06) -0.77(-1.25, -0.29) -0.84(-1.31, -0.38)	- 1.84 138.37		- 46% 88%	- 0.18 0.000	2.16 3.16 3.55	0.03 0.002 0.000
	POAG NTG	819 728	-0.31(-1.11, 0.48) -1.02(-1.54, -0.51)	46.62 12.99		87% 77%	0.000 0.005	0.78 3.91	0.44 0.000
The type of glaucoma	PCG GLC	631 170	-1.47(-2.12, -0.81) -0.90(-1.44, -0.36)	15.94 -		75% -	0.003 -	4.40 3.24	0.000 0.001
	EXG PACG PXSG	351 130 -	-1.14 (-2.34, 0.05) 0.70(-0.00, 1.40) -	15.27 - -		87% - -	0.000 - -	1.87 1.95 -	0.06 0.05 -
The type of machine	ORA Corvis ST ORA and Corvis ST	2680 - 139	-0.80(-1.20,-0.40) - -1.34(-1.95, -0.73)	139.97 - -		86% - -	0.000 - -	3.89 - 4.31	0.000 - 0.000

*No.* the number of eyes, *MD* mean difference, *CCT* central corneal thickness, *CH* corneal hysteresis, *CRF* corneal resistance factor

For the CH values, the subgroup analysis of ‘Age’ group, the heterogeneity was significantly reduced in the group of ‘ < 18 years old’(I^2^ = 0%). For treatment history, only ‘Used medicine’ group showed low heterogeneity(I^2^ = 0%). For the type of glaucoma, only the heterogeneity of ‘PCG’ group decreased significantly(I^2^ = 0%). For the CRF values, we conducted subgroup analyses according to the four aspects, the results all showed moderate or high heterogeneity(I^2^ > 25%). We did not include the IOPcc/g values, because we considered there was an inevitable relationship between IOP and glaucoma.

### Sensitivity analysis

**Fig. 7:**
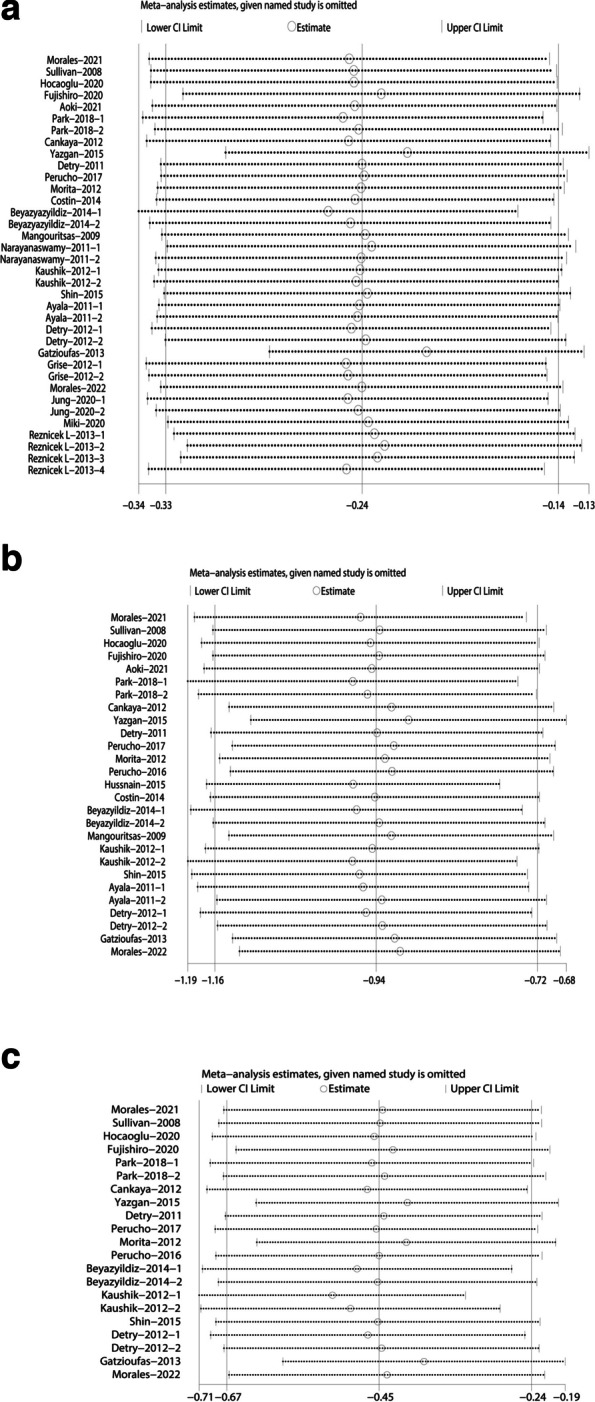
Sensitivity analysis summary. **a** Sensitivity analysis of CCT. **b** Sensitivity analysis of CH. **c** Sensitivity analysis of CRF

We conducted a sensitivity analysis to assess the stability of the results of the CCT, CH and CRF(heterogeneity: I^2^ > 50%) (Fig. 7), and the AL values were excluded because of the meaningless comparative result. The CCT, CH and CRF values’ comparisons were all in the effective range.

### Publication bias

We conducted publication bias tests for the outcome of CCT, CH and CRF (Table 3). The results using Egger’s test of CCT values and CRF values indicated that there was no significant publication bias(P_CCT_ = 0.459; P_CRF_ = 0.319). While the result of CH values showed that there was statistically publication bias in glaucoma and normal subjects(P_CH_ = 0.023).

**Table 3:** Publication bias of CCT, CH and CRF

	The *P* value of Egger’s Test
CCT	0.459
CH	0.023
CRF	0.319

### Quality assessment

All the studies we included were non-randomized studies, therefore we used the NOS items to assess the quality (Table 4). We evaluated the studies by examining 3 items: patient selection, comparability and outcome assessments. Studies were ranked according to the star scoring scale, with higher scores indicating higher research quality.

**Table 4:** Quality Assessment of Included Studies in the meta-analysis

Study	Patient selection	Comparability	Outcome assessments	Sum of score
Morales, 2021 [8]	****	*	**	7
Sullivan, 2008 [9]	****	*	**	7
Hocaoglu, 2020 [10]	****	**	**	8
Fujishiro, 2020 [11]	****	*	**	7
Aoki, 2021 [12]	****	**	**	8
Park, 2018 [13]	****	**	**	8
Cankaya, 2012 [14]	****	*	**	7
Yazgan, 2015 [15]	****	*	**	7
Detry, 2011 [16]	****	*	**	7
Perucho, 2016 [17]	****	*	**	7
Perucho, 2017 [18]	****	*	**	7
Gatzioufas, 2013 [19]	****	*	**	7
Morita, 2012 [20]	****	*	**	7
Costin, 2014 [21]	****	*	**	7
Beyazyıldız, 2014 [22]	****	*	**	7
Mangouritsas, 2009 [23]	****	*	**	7
Narayanaswamy, 2011 [24]	****	*	**	7
Kaushik, 2012 [25]	****	*	**	7
Shin, 2015 [26]	****	*	**	7
Ayala, 2011 [27]	****	*	**	7
Detry, 2012 [28]	****	*	**	7
Grise, 2012 [29]	****	*	**	7
Morales, 2022 [33]	****	*	**	7
Jung, 2020 [31]	****	*	**	7
Miki, 2020 [30]	****	*	**	7
Hussnain, 2015 [32]	****	*	**	7
Reznicek, 2013 [34]	****	*	**	7

## Discussion

Glaucoma is the leading cause of irreversible blindness. Most patients are diagnosed when they appeared clinical manifestations, while the lesions have already reached a certain degree at that time. Even though there has been a dramatic improvement in the prognosis over decades because of the introduction of new techniques, like new operative methods and trabeculectomy, the further improvements in clinical practice are still required. The follow-up of post-operative needs the support of new technology as well.

CH and CRF values were known to decrease with increasing age [35, 36], it has been reported that CH and CRF increased in eyes with large CCT as well. The reason was considered that a thicker cornea contained more ground substance and collagen fibers, which produce a higher damping capacity and resistance against deformation [11]. In the analyses, the results demonstrated that the CCT, CH and CRF values in glaucoma patients were statistically lower than that of normal subjects who in the relative age range of the same levels. The result of AL was not significantly different in glaucoma and normal subjects. Our analysis results agreed with the above conclusions, as CCT is positively correlated with CH and CRF. And the results also showed that the CCT, CH and CRF values of glaucoma patients were smaller than those of their peers. The data indicated that glaucoma might influence the corneal biomechanical characteristics.

In view of the influence of age factors, we took 18 years old as a dividing line, and compared subjects above and below 18 years old. The subgroup analysis results displayed that CCT, CH and CRF values significantly decreased in the ‘ ≥ 18 years old’ group, with the heterogeneity was slightly or prominently high. The data demonstrated that in comparison with peers, glaucoma patients older than 18 years old had more significant reductions in CCT, CH and CRF than those younger than 18 years old subjects. On the other hand, the conclusions obtained from the more stable corneal biomechanical characteristics of adults are more representative.

The effect of anti-glaucoma eye drops on corneal biomechanical properties is an influencing factor which needs to be considered. It has been reported that anti-IOP eye drops could change the corneal biomechanics [37–39]. In our subgroup analyses, the result showed that CCT was not significantly decreased, while the CH and CRF values were statistically decreased. However, the result still needs more future studies to verify because only one to three studies definitely indicated the treatment history of glaucoma patients they included.

Different types of glaucoma have different clinical symptoms and fundus manifestations, as well as corneal biomechanical characteristics. According to the subgroup analysis, which included 27 studies totally covered 8 kinds of glaucoma, the results demonstrated that there was no heterogeneity in CCT values of PACG. The CH values of all glaucoma types were all significantly lower than normal subjects except the PACG, and there was no significant heterogeneity of PCG, while others were all high. The included studies which contained the CRF, the NTG, PCG and GLC showed low CRF values compared with normal, however, it also showed high heterogeneity. The results indicated that different types of glaucoma caused different influences of CCT, CH and CRF.

Machine types and measurement means are common sources of error in the process of experiment and clinic. The most included studies used ORA as the measuring machines. And analysis results showed that the heterogeneity was all significantly high, the observation which used Corvis ST to detect the CCT demonstrated moderate heterogeneity, and none studies used Corvis ST to measure CH and CRF alone. Because there is not enough data from Corvis ST included, the conclusions we obtained need to be further verified.

## Conclusion

In this study, we conducted a meta-analysis to evaluate the accessibility between glaucoma and corneal biomechanical characteristics. In conclusion, the study provides that corneal biomechanical characteristics are associated with glaucoma, and the corenal biomechanics are different in various types of glaucoma. Corneal biomechanics can be a reference for the diagnosis of glaucoma, but it cannot diagnose glaucoma definitely.The findings of the study can provide some designed ideas of glaucoma screening, treatment, prognosis and related public health strategies.

## Data Availability

The data used and/or analyzed during the current study are available from the corresponding author on reasonable request.
